# Commentary: What will be the denouement for mobile extracorporeal life support?

**DOI:** 10.1016/j.xjtc.2022.02.002

**Published:** 2022-02-19

**Authors:** Ashish S. Shah

**Affiliations:** Department of Cardiac Surgery, Vanderbilt University Medical Center, Nashville, Tenn

**Figure:**
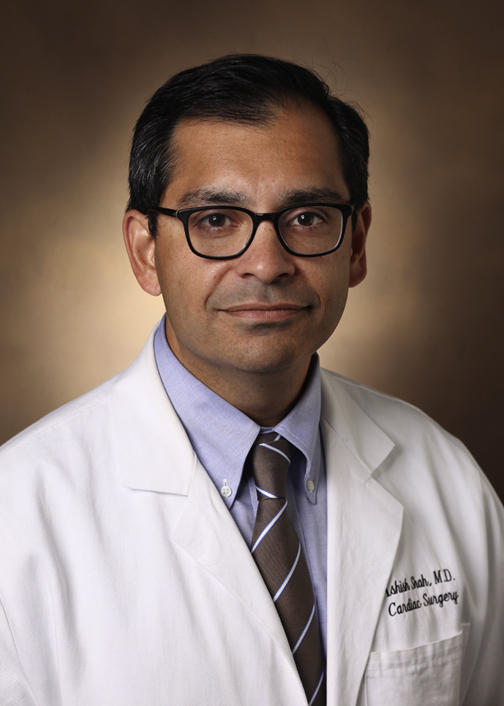
Ashish S. Shah, MD

**Central Message**

Mobile ECLS is an effective strategy in select patients. However, this story needs an ending.

See Article page 78.

All stories need a denouement: an ending, a finale. Gustav Freytag's pyramid is a construct for writers that tells us all stories need several components.^1^ Mobile extracorporeal life support (ECLS) is a compelling story that has a cast of interesting characters, innovation, struggles, bleeding, and a climax involving cannulation at the Louvre and pandemics. Freytag's next stage is called falling action. Here, the exciting climax results in various conflicts and new questions. Finally, the dramatist should offer a denouement or resolution. Despite Gustav Freytag's personal and alarmingly bigoted worldview, he has provided a useful framework to examine all dramatic work.

The current report from the group at the University of California Los Angeles details the climactic rise of a mobile ECLS program.^2^ The group admirably develops a ground-based team that offers cannulation and transport back to University of California Los Angeles, with satisfactory outcomes. Their experience mirrors what multiple mobile ECLS teams around the world have previously reported over the last 20 years.^3^ What remains is the critical resolution. We can put people on ECLS and transport them anywhere in the world, but we don't know *how* we should triage this complex therapy. We don't know *how* we should organize this on a regional, national, and international level. *How* should we address liability, professional standards, and which institutions should be primarily responsible? Finally, given the capacity constraints in even wealthy Western health systems, who should pay for all this running around? The current report fails to shed any new light here. Noticeably absent are the details of patients who were turned down by their team and, importantly, why. The variety of complications also remain distressingly high and are similar to other studies. So, how does this story conclude? Certainly, as the current report confirms, mobile ECLS is a valid platform to save lives. How we ultimately resolve the problematic issues, questions, and conflicts that it creates is how this story ends.

## References

[1] Glatch S. The 5 elements of dramatic structure: understanding Freytag's pyramid. Writers.com. https://writers.com/freytags-pyramid.

[2] Hadaya J., Sanaiha Y., Gudzenko V., Qadir N., Singh S., Nsair A. (2022). Implementation and outcomes of an urban mobile adult extracorporeal life support program. J Thorac Cardiovasc Surg Tech.

[3] Tipograf Y., Liou P., Oommen R., Agerstrand C., Abrams D., Brodie D. (2019). A decade of interfacility extracorporeal membrane oxygenation transport. J Thorac Cardiovasc Surg.

